# Detection of Microorganisms in Granulomas That Have Been Formalin-Fixed: Review of the Literature Regarding Use of Molecular Methods

**DOI:** 10.6064/2012/494571

**Published:** 2012-12-31

**Authors:** Jeannette Guarner

**Affiliations:** Department of Pathology and Laboratory Medicine, Emory University, Atlanta, GA 30322, USA

## Abstract

Granuloma is an organized aggregate of immune cells that under the microscope appear as epithelioid macrophages. A granuloma can only be diagnosed when a pathologist observes this type of inflammation under the microscope. If a foreign body or a parasite is not observed inside the granuloma, stains for acid-fast bacilli and fungi are ordered since mycobacteria and fungi are frequently the cause of this type of inflammation. It is calculated that 12 to 36% of granulomas do not have a specific etiology and many have wondered if with new molecular methods we could reduce this number. This paper will summarize the frequently known causes of granulomas and will present the recent literature regarding the use of molecular techniques on tissue specimens and how these have helped in defining causative agents. We will also briefly describe new research regarding formation and function of granulomas and how this impacts our ability to find an etiologic agent.

## 1. Introduction

Granuloma is an organized aggregate of immune cells that under the microscope appear as epithelioid macrophages [1]. Epithelioid macrophages are enlarged phagocytic cells that have abundant cytoplasm and can sometimes coalesce to form multinucleated giant cells. The epithelioid macrophages and giant cells are accompanied by other inflammatory cells including lymphocytes, plasma cells, and polymorphonuclear leukocytes as well as varying degrees of necrosis. The process may be confined forming a granuloma or can infiltrate surrounding tissue thus being called granulomatous inflammation. This chronic inflammatory response has been attributed to delayed hyperimmune reaction to a persistent noxious stimulus. Traditionally, granulomatous inflammation is thought to wall of the noxious agent. However, new experimental observations in the formation of granulomas are now emerging and giving new insides as to how they form and if in reality they are encasing the agent that causes them.

A granuloma or granulomatous inflammation can only be diagnosed when a pathologist observes this type of inflammation under the microscope. If a foreign body or a parasite is not observed inside the granuloma, stains for acid-fast bacilli (AFB) and fungi (GMS) are ordered as mycobacteria and fungi are frequently the cause of this type of inflammation. There are other diagnostic entities that need to be entertained depending on the tissue, but it is customary to rule out mycobacteria and fungi first before considering other diagnostic possibilities. It is calculated that 12 to 36% of granulomas do not have a specific etiology [2, 3] and many have wondered if with new molecular methods we could reduce this number. This paper will summarize the frequent known causes of granulomas and review the recent literature regarding the use of molecular techniques on tissue specimens that have helped in diagnosing the cause. We will also briefly describe new research regarding formation and function of granulomas and how this impacts our ability to find an etiologic agent.

## 2. Causes of Granulomas

From the morphologic perspective, granulomas can present different amounts of necrosis, different sizes and shapes (stellate or geographic), different amounts and types of accompanying inflammatory cells (suppurative, plasma cells), or the macrophages may show some characteristic morphology (foamy macrophages). Tuberculosis is the most frequent cause of granulomatous inflammation worldwide. Other infectious causes include fungi (histoplasmosis, cryptococcosis, blastomycosis, coccidioidomycosis and some of the molds), parasites (schistosomiasis, leishmaniasis, fasciolosis, enterobiasis), and other bacteria (non-tuberculoid mycobacteria, cat scratch diseases—bartonellosis, tularemia, syphilis, listeriosis, Q fever). Viruses are rarely mentioned as causes of granulomas. Some of the morphologic characteristics tend to be associated with specific organisms and Table 1 presents a correlation between different granuloma morphologies and the usual etiologic agent encountered [2, 4]. 

The correlation of a specific organism with a characteristic granuloma morphology can vary, particularly in immunosuppressed individuals. For example *Bartonella henselae* can cause cat-scratch disease which is characterized by stellate granulomas but in immunosuppressed patients this bacteria can cause bacillary angiomatosis-peliosis [5]. In HIV infected patients, cutaneous histoplasmosis can have mononuclear and plasma cell inflammatory infiltrate in the superficial and deep dermis with perivascular, perifollicular, and lichenoid patterns and not a granulomatous inflammatory pattern [6]. Although granulomatous inflammation is the usual morphology described for patients with *Mycobacterium tuberculosis*, there have been some descriptions of cases in which the inflammatory response is not granulomatous. A study of necrotizing nongranulomatous lymphadenitis showed presence of *M. tuberculosis* in 6 out of 35 cases by using molecular methods in formalin-fixed, paraffin-embedded tissues [7]. 

Granulomas or granulomatous inflammation due to an infectious agent can be associated with other pathologies; thus it may be difficult to recognize it as independently caused entities. The collision of two entities has been documented in HIV patients that have in the same lesion Kaposi sarcoma and granulomas with presence of acid-fast bacilli and molecular evidence of mycobacteria [8]. Patients with breast cancer and tuberculosis in the same lymph node have been described [9]. 

In some instances there is a granuloma and an infectious agent is found; however, this is not a typical organism that causes granulomas such as *Pneumocystis* [4, 10]. In these instances the pathologist wonders if other causes should be searched for; however, review of the literature of cases with *Pneumocystis* and granulomas has not shown presence of other organisms. The variable histopathologic pattern in the presence of the same organism suggests that host factors are at play [11, 12]. The host factors that have been proposed include presence of CD4 and CD8 lymphocytes, previous *Pneumocystis* exposure, absence of IgA antibodies against *Pneumocystis*, and the exposure time to the fungal glycoproteins. The role played by the immune system in formation of granulomas is further emphasized when considering some congenital immunodeficiencies. The typical example is chronic granulomatous disease which is a childhood disease involving defects in the phagocytic function as there is reduced nicotinamide adenine dinucleotide phosphate (NADPH) oxidase complex. These patients mount granulomatous inflammation towards most infectious agents and even noninfectious etiologies [13]. Other immune deficiencies such as common variable immune deficiencies can also present granulomatous inflammation towards infectious agents that are not usually associated with granulomas [14].

There are noninfectious causes of granulomas. Foreign bodies such as talc, beryllium, mineral oils, lipids, or medications can produce granulomatous inflammation in diverse organs. Sarcoid-type granulomas are also seen in lymph nodes of patients with lung, stomach or uterine neoplasias [15–17] or may appear in the skin preceding or concomitant with the initial diagnosis of cancer [18]. The presence of granulomatous inflammation may confer better prognosis in some neoplastic diseases as it may indicate some degree of host antitumor activity. This has been documented in patients with small cell carcinoma of the lung in whom there is sarcoid-like granulomatous inflammation of regional draining lymph nodes [19]. Hodgkin's disease is the neoplasia most frequently cited to cause diagnostic confusion because of multisystem granulomatous inflammation which can have various degrees of necrosis [15]. A variety of immune dysfunctions associated with the neoplasia such as a systemic inflammatory reaction with production of chemokines, interleukins and angiogenic factors, immune cellular changes such as anergy, and epigenetic changes such as hyper-methylation have been implicated in the formation of the granulomatous inflammation [18]. Nowadays with the use of immunomodulators such as interferon-*α* and monoclonal antibodies for different neoplasias and other infections, granulomatous inflammation has been seen after these treatments with no evidence of an infectious agent [20, 21]. 

There are entities that cause granulomas and are specific for certain organs. For example, in the upper respiratory tract Wegener's ganulomatosis, Churg-Strauss syndrome, or cocaine-induced midline destructive lesions [22] need to be considered. In the liver the entities that need to be considered include primary biliary cirrhosis, primary sclerosing cholangitis, hepatitis C, and others [2, 3]. Also to consider is the variable distribution of granulomas in different tissues in the various entities. For instance, although we think of sarcoid as having most of the lesions in skin, respiratory tract, and lymph nodes [23], 80% of patients have been shown to have microscopic hepatic granulomas even though symptomatic liver disease is not frequent [24]. From the pathologic perspective we think of *Brucella*-associated hepatic fibrin ring granulomas; however, these are only seen in 2 to 10% of cases [2] while vertebral and osteoarticular involvement is probably much more common and the usual causes of mortality include endocarditis and central nervous system involvement [25, 26]. 

 The frequency of the different etiologies for the granulomatous inflammation will depend on the prevalence of the diseases in that particular geographical area. For example, a study in Southern Iran of causes of liver granulomas showed that a little over 50% of their cases were due to tuberculosis and 8% were due to visceral leishmaniasis while other etiologies included fungal infections, visceral larva migrans, primary biliary cirrhosis, hepatitis C, cytomegalovirus, lymphoma, and others [3]. In this study they used a variety of methods to define the causative agent including histopathology with special stains, PCR, autoantibodies, viral markers, and other characteristics obtained from the chart review which were not always evident at the time the hepatic biopsy was studied. A study in Turkey of hepatic granulomas found that 44% of their patients had different stages of primary biliary cirrhosis, while only 12% had tuberculosis (all 10 patients diagnosed with PCR and one having AFB positive staining), 6% had neoplasias (primarily cancers of hepatic origin), and 4% had sarcoidosis [27]. An Egyptian study of cutaneous granulomas showed that tuberculosis accounted for 40% of cases followed by leprosy (32%), leishmaniasis (16%), while actinomycetes and fungi were rare [28]. Diagnostic methods for the Egyptian study included intradermal tests, biopsy, serology, direct smear from lesion and PCR.

## 3. *Mycobacterial* Detection Using Molecular Methods in Patients with Granulomatous Inflammation 

Mycobacterial DNA can be recuperated and the species identified using molecular methods in a variety of samples such as sputum, cultures, formalin-fixed, paraffin-embedded tissue blocks that have been used for histopathology, and bones of mummies from Egyptian times [29]. Different primers have been used including: (a) the 123-base pair (bp) segment of the repetitive IS6110 sequence of *M. tuberculosis *complex, which covers *M. tuberculosis*, *M. bovis*, *M. microti*, *M. africanum*, and *M. canettii* [30–32], (b) EUB338 [33], (c) MTB187 [33], (d) MAVP187 [33], (e) 16S rDNA [34], (f) hsp65 [35], (g) KD1 and KD2 [36], (h) 16SOL-16SOR and 16SIL-16SIR [37], and (i) *pncA *(Rv2043c) [38]. For the most part, DNA from different specimens is extracted then PCR reactions are performed to detect mycobacteria. Confirmation that the product is* M. tuberculosis* or another mycobacteria has been done by a variety of methods including sequencing and Southern blot. Other methods that have been used to test cultures or formalin-fixed, paraffin-embedded tissue sections are peptide nucleic acid fluorescent in situ hybridization [39], rRNA oligonucleotide probes [33], and spoligotyping (a hybridization step after IS6110 PCR amplification that allows differentiation of the nonrepetitive DNA spacers of *M. tuberculosis* and *bovis*) [32].

Immunohistochemistry has been used to detect mycobacterial antigens in tissue sections and some authors have found that this technique has better sensitivity than molecular methods in addition to being easier to perform in resource poor settings [40]. However, with immunohistochemistry there is cross-reactivity between the different mycobacteria species [41]. 

It is important to realize that in many occasions a tissue sample is not sent for cultures since a granuloma or granulomatous inflammation is not found until microscopy is performed and all the tissue, by then, has placed in formalin. Also, a sample may have been sent for culture but neither mycobacteria nor fungi grew from the specimen sent to microbiology, thus the only material available for testing is the formalin-fixed, paraffin-embedded tissue. Following we will describe the studies that have focused on studying formalin-fixed, paraffin-embedded material exclusively.

1. A study of 48 formalin-fixed, paraffin-embedded tissues with granulomas in lung, bone, lymph nodes, liver, genitourinary tract, and other tissues showed positive AFB staining in 8 (17%) cases and molecular evidence of mycobacteria in 19 (40%) [32]. The authors used the repetitive IS6110 sequence of *M. tuberculosis* complex to define the presence of mycobacteria and spoligotyping was used to differentiate between the different species. The authors identified *M. tuberculosis* in 7 cases, *M. bovis* in 4 and in 9 cases the spoligotypic was not able to define the mycobacteria present. Of the cases that were AFB positive, *M. tuberculosis* was found in 5 cases, *M. bovis* in 1, and in 2 the determination of the mycobacterial species was not possible.
2. Another study of 190 cases with granulomas from the respiratory tract, lymph nodes, and other tissues targeted the *hsp65* gene in a nested PCR to detect all mycobateria then sequenced or used restriction fraction polymorphisms for species identification [35]. In some cases culture results were available and concordance of molecular and culture results was found in 83% (34/41 cases). A total of 119 (63%) cases were positive for mycobacterial DNA. The typical restriction pattern for *M. tuberculosis* complex was present in 71 cases (60%) while non-tuberculous mycobacteria were found in 41 cases (34%) and identification was not possible in 15 cases (36%). Only 7 cases showed AFB positive staining out of 14 that had *M. tuberculosis* by culture and molecular methods.
3. In a study from Thailand of 120 skin cases with granulomas in which cultures had been obtained, PCR using 16S rRNA common fragment for mycobacteria and a second 306 bp DNA fragment specific for *M. tuberculosis* was performed [37]. AFB staining in formalin-fixed, paraffin-embedded sections showed positivity in 31%, by PCR 36% were positive and by culture 30%. There were 12 cases that were positive with both AFB stains and PCR, 21 that were AFB positive but PCR negative, and 35 that were PCR positive but AFB negative. The sensitivity and specificity of PCR against AFB results was 29 and 61%, respectively, and when compared to culture these were 67 and 76%. The low number of cases with positive results for PCR was attributed to inhibitors present in the tissues.
4. A study from Hong Kong of 115 patients correlated histopathology, culture and PCR results [30]. The authors used the IS6110 target and confirmation of *M. tuberculosis* was performed by Southern blot hybridization. Between 1 to 3 paraffin blocks per patient were tested as they assumed that mycobacteria were scant and randomly distributed in the tissues. They looked at granulomatous inflammation of 62 pulmonary specimens and 53 extrapulmonary sites. Only 68 patients had PCR and culture results. The authors showed that 20 patients were positive using culture and PCR, 2 were PCR negative but culture positive, and 13 were negative for culture and PCR. An additional 35 cases were positive with PCR but no cultures were available for them. Positive AFB staining and PCR occurred in 12 cases.
5. A study from Korea looked at 250 patients with culture and AFB smears that required lung biopsies because the clinical diagnosis of tuberculosis was uncertain [31]. In this study the authors calculated sensitivity and specificity of nested PCR using the IS6110 target against culture to be 85 and 99%, respectively.
6. A study of 50 Indian patients with abdominal tuberculosis showed 31 cases positive with histopathology and 30 with a PCR that used the KD1 and KD2 target [36]. Only 24 cases were concomitantly positive with histopathology and PCR and 11 that were negative with both techniques. In 4 patients the PCR did not yield results due to inhibitors.
7. A study of 12 Japanese patients with granulomatous inflammation of musculoskeletal tissues used the 16S rDNA and sequenced the products of the formalin-fixed, paraffin-embedded tissues [34]. They obtained amplicons from 5 cases (41%) and the mycobacteria found included *M. tuberculosis* in 4 cases and *M. avium* in one. Four of these cases showed AFB in the tissues, 3 cases had *M. tuberculosis* and one *M. avium*.
8. A second group from Korea correlated histopathology, AFB staining and a nested PCR using the repetitive IS6110 sequence of *M. tuberculosis* complex in 81 patients [42]. They studied a variety of tissues from the respiratory tract, lymph nodes, gastrointestinal tract, soft tissues, and others. They had 53 cases with granulomatous inflammation in which 17 were AFB positive and 36 PCR positive. Of the 17 AFB positive cases 14 were also PCR positive thus 3 cases were attributed to other mycobacteria. Of the 28 cases with chronic inflammation but no definitive evidence of granulomas none showed positive AFB while 10 showed evidence of *M. tuberculosis* by PCR.
9. A study from Spain of 10 patients with papulonecrotic tuberculid skin lesions demonstrated that 8 had *M. tuberculosis* complex in formalin-fixed, paraffin-embedded skin biopsies [43]. The authors used a 285-bp sequence specific of *M. tuberculosis* complex that was confirmed by Southern blot hybridization. In this study the patients did not have respiratory evidence of tuberculosis but all were PPD positive.

Although these studies have used an array of different clinical presentations, tissues, and pathologies, they have established that PCR can detect mycobacteria in 36 to 60% of formalin-fixed, paraffin-embedded specimens. In many instances staining AFB in the tissue was not observed. If the primer used in the study was specific for *M. tuberculosis*, presence of AFB staining did not correlate with PCR positivity and in those cases the authors commented that the AFB could correspond to other mycobacteria. From the previous studies that used formalin-fixed, paraffin-embedded tissues and others that have used a variety of materials such as fresh samples and cultures it is clear that obtaining a product for PCR analysis using a broad mycobacterial target then sequencing and comparing with known GenBank sequences can reveal that those mycobacteria are not *M. tuberculosis* but other mycobacteria. Examples of detection of these mycobacteria are presented in Table 2. 

Detection of mycobacterial species does not seem to improve if fresh tissues rather than formalin-fixed, paraffin-embedded tissues are used. A study from India that used fresh tissue to perform PCR for *M. tuberculosis* targeting the IS6110 gene and compared to histopathology (necrotizing granulomas) showed that 20 of 104 (19%) samples were both positive with AFB and PCR while 74 (71%) were negative for both [44]. Discrepant results occurred in 7 cases, 4 of these were positive with the AFB stain but negative with PCR and 3 were positive with PCR but negative with AFB.

Molecular techniques are expected to increase sensitivity and specificity; however, in some instances this does not occur. A study of bovine lymph nodes with *Mycobacterium bovis* used the IS*1081* which detects down to 1 genome copy and the RD4 which detects 5 genome copies. The authors determined a sensitivity of 70% and 50%, respectively, for each probe compared to culture [45]. When analyzing why the recovery with PCR was lower than expected, the authors determined that there was limited recovery of mycobacterial DNA which may have been due to the resilient mycobacterial cell wall, the presence of tissue debris and the paucibacillary nature of some of the lesions. In order to decrease the amount of tissue debris researchers have compared PCR on laser capture microdissection material to the entire formalin-fixed, paraffin-embedded tissue sections but found no improvement in sensitivity and specificity for detection of *M. tuberculosis* [46].

 In some instances the organism detected by the molecular technique may not be the same obtained in cultures. A study of resected lung specimens from 24 patients with non-tuberculous mycobacterial disease correlated the organisms identified by culture of tissue or sputum to the results obtained from molecular testing using IS6110 to detect *M. tuberculosis* and hsp65 gene which was later sequenced [47]. In 12 patients the culture and sequencing analysis rendered the same organism. In 8 patients the culture and PCR results were discrepant. In 6 of the patients the culture showed a rapid grower. Ten of the patients had fibrocavitary disease and in all but 2 more than one mycobacteria were identified. The authors suggested that the frequent discrepancies between the PCR and culture results were possibly due to multiple non-tubercoulus bacteria colonizing the lesions. The authors comment that when using PCR, the most abundant mycobacteria is the one identified although in some instances more than one mycobacteria were observed. In contrast, the organism that overtakes the culture is the one growing faster. 

By use of molecular techniques new species of mycobacteria are being discovered. For example, a case of skin papules and nodules that showed granulomatous inflammation in a patient with Hodgkin's disease and severe cellular immunodeficiency demonstrated *Mycobacterium simiae* when using the sequence of the product of 16S rRNA. However, based on culture characteristics, susceptibility testing, molecular testing targeting different portions of the mycobacteria gene (*rpoB *and *hsp65*), and high-performance liquid chromatography of mycolic acid methyl esters the mycobacteria was determined to be a novel one and the authors proposedthis to be called *M. shigaense* [48].

Molecular testing has also been useful in study of nosocomial infections as it can define the species of mycobacteria and other mutations that can occur. For example, random amplified polymorphic DNA-PCR was used to define clonicity of *Mycobacterium abscessus *in isolates from 8 patients that had granulomatous prostatitis after biopsies had been performed on them [49]. All isolates were indistinguishable from one and other suggesting that all isolates came from the same clone and had been nosocomially acquired. 

## 4. Detection of Organisms Other than Mycobacteria Using Molecular Methods in Patients with Granulomatous Inflammation

Our currently used culture and staining techniques are optimized to identify microbial agents that are known to cause disease. Similarly, detection of antigens, antibodies, or presence of nucleic acids are targeted to identify a particular suspect disease.  Table 3 presents methods that can be used for diagnosis of infectious agents not related to mycobacteria that cause granulomatous inflammation. 

To discover new pathogens, molecular methods that use conserved portions of microbial genomes to obtain products which are then sequenced are ideally suited for this task. For bacteria and fungi, some portions of the ribosomal nucleic acids are highly conserved and can be used to determine the genus and species. *Tropheryma whippelii* is a clear example of discovering an agent for a disease that had long been thought to be infectious but in which cultures had failed to give us the microbial agent [97]. The discovery was based on obtaining a product using a 16S ribosomal RNA (rRNA) primer and then sequencing the product. Sequencing the 16S rRNA product of bacillary angiomatosis lesions demonstrated that *B. henselae* (previously *Bartonella bacilliformis*) was the causative agent [98]. In the case of *T. whippelii* the genetic sequence was not recognized thus this was a new distinct bacterium while comparing the sequence of *B. henselae* to others published at the time demonstrated that the bacteria present in bacillary angiomatosis lesions was related to *Bartonella *and *Rochalimaea*. As described previously, this conserved nucleic acid sequence of bacterial small rRNA has been used for detection of different mycobacteria species. In the case of fungi, products of conserved rRNA genes (18S, 28S and 5.8S) or the intervening internal transcribed spacer (ITS1 and ITS2) regions can be obtained, sequenced and the latter compared to what has been published to help determine the fungal genus and species present in the tissue [84, 99, 100]. A retrospective study of fungi in formalin-fixed, paraffin-embedded tissues using conserved primers and sequencing the product obtained showed that nucleic acid recovery varied from 60 to 90% depending on the method used for extraction and led to a PCR efficiency of 57 and 93% which translated to approximately 60% recovery of the fungi to the genus level [101]. For parasites and viruses, widely conserved regions are not available.

Even with these advances, we are constantly confronted with cases in which an infectious agent is suspected but no specific agent can be found. A typical entity falling in this category is sarcoidosis which has always been suspected to be of infectious origin but in which finding the causative organism has been elusive [23]. These patients have granulomas with little necrosis and no mycobacteria or fungi are observed in the tissue by using conventional techniques or evidence of a microorganism is found by a variety of methods (culture, skin testing) at the time of original diagnosis. New molecular methods and immunologic assays have been used to study a variety of sarcoidosis samples and the association with a variety of mycobacteria appears to be strong [23, 102]. Using *Mycobacterium* species 16S rRNA and *rpoB* sequences, researchers have found that 60% of lung biopsies and lymph nodes pathologically classified as sarcoid contained mycobacteria; however, the IS*6110 *specific for *M. tuberculosis* was negative [103]. This study found a heterogeneous group of mycobateria that are closely related to *M. tuberculosis*, *M. gordonae*, and *M. kansasii*. The amount of mycobacteria using real-time quantitative PCR targeting the *IS986* of the *M. tuberculosis* genome in formalin-fixed, paraffin-embedded specimens of patients with sarcoidosis is present in low numbers compared to controls [104]. Moreover, IgG antibodies against *M. tuberculosis* catalase-peroxidase have been found in a high proportion of patients with sarcoidosis compared to controls [105]. 

In addition to mycobateria, other organisms including *Propionibacterium acnes* have been found in greater amount in specimens from patients with sarcoidosis by molecular analysis too [23, 106]. The literature that links mycobacteria with sarcoidosis has primarily been observed in American subjects while that linking *Propionibacterium *with sarcoidosis is in Japanese persons, suggesting that host genetics play an important factor regarding the possible causative agent to what we now call sarcoidosis. Even when organisms, in particular mycobacteria, are isolated or found in patients in whom a previous diagnosis of sarcoidosis had been given, the question of whether the mycobacteria was the cause of the granulomas or if there are concomitant diseases (sarcoidosis and an infection with a mycobacteria) is always present [102].

Nowadays a revolution in microbiology laboratories is taking place with the use of matrix-assisted laser desorption/ionization time-of-flight (MALDI-TOF) mass spectrometry analysis [107, 108]. This method studies the composition of biomolecules in bacteria including proteins, DNA, sugars and others. The fingerprint of a growing number of bacteria are now in the libraries of MALDI-TOF mass spectrometers. For this method, bacterial colonies are placed in a matrix and allowed to cocrystallize with the matrix as it dries. Then the sample is fired with a laser causing ionization of the biomolecules. The ionized particles are pulsed into a time-of-flight mass spectrometer. Time-of-flight mass spectrometers have a “mirror” that reflects the ions and allows for better discrimination between the biomolecules measured creating a fingerprint pattern specific for the bacteria. Then the pattern of the suspect organism is compared to a library of patterns and identification to the genus and species level can be achieved based on the specific fingerprint of biomolecules. For some organisms the discrimination of the fingerprint has a success that ranges between 89 to 100% for the genus and between 84 to 95% for the species. Since identification is based on the organisms available in the library, as a wider diversity of organisms are entered into the libraries of the different instruments in the market identification rate will improve. At this point, the instrumentation is expensive; however, the identification of each sample is cheap. Bacterial identification is much faster once a pure colony has been isolated since bacterial growth with production of a variety of biochemical reactions is not needed which will be a major improvement in turn-around time for identification of mycobacteria. It should be noted that for some organisms an extraction phase is necessary. Study of primary specimens using MALDI-TOF is being pursued for sterile sites such as blood, cerebrospinal fluid, and fine needle aspirates which may allow discovery of microbiologic agents that are fastidious growers and cause granulomatous inflammation.

Lastly, there may be some granulomatous inflammatory processes in which a causative agent may never be found and some authors have suggested that host factors play a mayor role. A Canadian study of central nervous system granulomas with caseous necrosis but in which an etiologic agent could not be determined, demonstrated no improvement or worsening of clinical and radiologic features after treating with antimycobacterial regiments but improvement after immunomodulation therapy with prednisone or azathioprine [109]. In this study, search for causative agents, primarily mycobacteria, included a variety of stains, cultures, and PCR and the authors suggested that if no organism is found the most probable cause is not infectious but a variant of a hypersensitivity reaction, possibly sarcoidosis, where granulomas show necrosis. 

## 5. Formation and Function of Granulomas

 Traditionally granuloma formation has been thought as an adaptive cellular immune process. There are several phases in the formation of granulomas: initiation, accumulation, effector, and resolution [24]. Initiation takes place when an antigen is presented by a macrophage or dendritic cell to CD4 lymphocytes. The presentation is usually done through use of Toll-like receptors that recognize a variety of structural components such as mycolic acids and peptidoglycans present in the cell wall of mycobacteria and other granuloma inducing agents [110]. T helper 1 (Th1) CD4 cells are usually involved in mycobacterial diseases and sarcoidosis, while T helper 2 (Th2) CD4 cells are usually involved when there is a parasitic infection. As infection progresses, there can be switching from Th1 to Th2 cell response. Th1 cells produce Il-2 and interferon *γ* (IFN*γ*) which recruit more CD4 cells and macrophages and are indispensible for the accumulation phase [110]. Other chemokines that play a role include tumor necrosis factor (TNF), and several interleukins produced by Th2 cells (IL-4, IL-5, and IL-13) and macrophages (IL-12). The Th1 response induced delayed hypersensitivity reactions while the Th2 response stimulates antibody production, in particular IgE [111]. The new milieu recruits CD8 lymphocytes and neutrophils and is important for the effector phase. Lastly the resolution phase includes the deposition of collagen and creation of fibrosis. This conventional thought regarding the formation of granulomas results in walling off the noxious agent.

 Recent experiments have challenged some of the concepts regarding the production of granulomas. Following we will explore how our understanding of different components has evolved with respect to granulomas formed by different agents. Neutrophils in granulomatous inflammation have an active role [112]. Experiments using intradermal injections of Bacille Calmette-Guerin (BCG) have shown that neutrophils, rather than macrophages, are the cells that carry the mycobacteria into the lymph nodes. Once in the lymph nodes, neutrophils present mycobacterial antigens to dendritic cells that then recruit CD4 cells and lead to production of IFN*γ*. In addition, neutrophilic proteins are present in macrophages located in ganulomatous inflammation. It is postulated that these proteins serve to enhance phagocytic properties of macrophages. Although the important role of neutrophils is histopathologically confirmed in *Bartonella, Listeria, Candida*, and *Toxoplasma* granulomatous inflammation [58], neutrophils have not been traditionally considered an important component in mycobacterial infections. 

Other researchers have demonstrated that innate immunity is an important component of granulomatous inflammation. Using zebrafish larvae which do not have adaptive immunity, researchers have shown that granulomas form when they are exposed to *Mycobacterium marinum* [1]. The zebrafish experiments have also demonstrated that macrophages that have ingested the infectious agent during early infection are responsible of propagating the infection to other macrophages as they undergo apoptosis rather than containing the infectious agent [113]. Macrophages forming granulomas move rapidly towards infected macrophages and ingest mycobacteria. This exchange usually occurs at the periphery of the granuloma. The signal for macrophage movement is derived from the pathogen and the dying infected macrophage. As the newly recruited macrophage ingests the contents of the dying one, it allows for mycobacteria to multiply and disperse with the moving macrophage. In this manner, the infection is not walled off but disseminated from a primary site to a secondary one. In addition to macrophages moving fast through the granuloma, T cells are also moving. Thus, it is postulated that there are 2 types of functional granulomas: those with rapid moving macrophages which are formed in response to infectious agents such as mycobacteria and those with slow moving macrophages which include those responding to foreign bodies [1]. 

 Review of histopathologic descriptions of tuberculosis in the preantibiotic era comment that primary infection resulted in an exudative reaction which included foamy alveolar macrophages and some degree of lipid necrosis and systemic dissemination of the mycobacteria [114]. Using molecular techniques, some investigators have corroborated that mycobateria are disseminated through the body by using PCR and spoligotyping [115]. The mycobacteria have been found during latent infection in vertebrae [29, 116], spleen, kidney and liver [115]. By using in situ hybridization, the mycobacteria have not only been present in macrophages, but also in endothelial cells of these organs [115]. Of interest is that in the same patient more than one bacterial genotype was found. 

Our understanding of the role that different mediators have has also evolved through time. For example, it appears that TNF is not indispensable for the formation of granulomas due to mycobacteria in zebrafish. However, the role of matrix metalloproteinase 9 (MMP9) secreted by epithelial cells is very important for recruiting macrophages and forming granulomas [117]. MMP9-knockout mice that have been infected with *M. tuberculosis* have poor granuloma formation and low amounts of mycobacteria. 

Besides the host factors discussed, there are new discoveries that involve mycobacterial factors. For example, it is now recognized that there are several cell wall lipids that arrest the maturation of phagosomes and do not allow acidification [118]. The shift from an external pH of 7 to the phagosomal pH of 6.4 shapes the transcriptional response of mycobacteria allowing them to continue to grow inside the macrophage by using host cholesterol and other cell lipids as nutritional sources. In addition, mycobacteria avoid destruction by superoxide by using superoxide dismutase and by cell wall lipidoglycans scavenging the oxygen radicals. The mycobacteria residing inside the phagosomes release proteins and lipids that traffic actively through the infected cell. As a pathologist one would expect abundance of bacilli in these lesions but the organisms are usually difficult to find. One explanation to pathologist's inability to find mycobacteria may be due to an incompletely formed cell wall during the replicative process which does not allow visualization with the techniques we currently use. Some researchers have studied the lipid composition of the caseous necrosis in tuberculoid granulomas and have found that there is dysregulation of lipid metabolism in macrophages with disproportionate abundance of certain lipid metabolic pathways [119] which explains why there is a lot more caseum than bacteria in them. Another possible explanation is that mycobacteria become cell wall deficient due to the host immune response and the shape and acid-fast staining of the mycobacteria is atypical or lacking [120, 121].

Microscopically, acid-fast bacilli are usually found in the interface between necrotic cellular debris and areas with caseous necrosis. As the granuloma becomes older, bacteria become sparser in the areas of hard necrosis. Review of histopathologic descriptions of tuberculosis in the preantibiotic era comment that post primary tuberculosis lesions start with foamy macrophages that have ingested the organisms [114]. In 90% of the cases the lesions regress spontaneously and fibrosis is noted in the area where the foamy macrophages were, but in 10% of the cases the lesions evolve to caseous necrosis and cavity formation. The numbers of acid-fast bacilli in these cavities are increased and as the individual coughs, other persons are at risk of acquiring the disease. 

A similar distribution of mycobacteria has been found in granulomas of immune competent patients caused by *Mycobacterium avium* complex [122]. During initial infection, when only one exudative lesion is seen, there are many organisms inside mononuclear cells. As the lesions progress to a well defined granuloma with necrosis, there are less organisms which are usually present in the periphery either inside mononuclear cells or in the necrotic material.

*Schistosoma* produced granulomas in humans show a mixed Th1-Th2 response that will vary depending on the genetic background of the host, nutritional status, intensity and duration of the infection, and concurrent pathology [111]. In murine animal models the Th2 response with a large population of eosinophils dominates the response to schistosome eggs. The Th2 response with production of IL-10 and IL-13 favors fibrosis but, this response may also depend on other compounds such as nitric oxide synthase. The Th1 response with production of TNF, IFN*γ* and IL-12 is responsible for antifibrotic activity [111, 123]. Also in play is an increase in Treg cells that modulate the Th1-Th2 response. In immunocompetent hosts, *Schistosoma *eggs elicit a collagen-rich granulomatous response to try to sequester egg products, but this same response can lead to extensive hepatic fibrosis. Understanding the pathobiology of this parasitic infection gives us insights into the difficulties that can occur when in a liver biopsy, granulomas with eosinophils and extensive fibrosis are found but no egg parasite can be identified. To further define what is causing the disease in each individual patient requires epidemiologic information that will guide further serologic, molecular and other studies.

Studies of formation of granulomas in vitro using peripheral blood cells from patients with acute and chronic Q fever and healthy controls have corroborated some of the findings from the zebrafish and shed some insights into differences encountered at various stages of the disease [124]. In their experiments, monocytes from healthy donors were the ones that initiated granuloma formation but well formed granulomas required the presence of T lymphocytes. In addition to monocytes and lymphocytes, their granulomas had epithelioid macrophages, and multinucleated giant cells, but no dendritic cells. Distance and trajectories covered by monocytes and T lymphocytes of healthy donors to *Coxiella* coated beads seems to be similar to that found in zebrafish; however, these were diminished if the monocytes and T lymphocytes came from patients with chronic Q fever. In addition, monocytes and T lymphocytes from chronic Q fever were either unable or displayed partial granuloma formation. The authors comment that their experiments recapitulate what happens in vivo since patients with acute Q fever form fibrin ring granulomas but as the infection becomes chronic the granulomas are defective and only spotty necrosis surrounded by collections of lymphocytes are observed. 

## 6. Conclusions

 Granulomatous inflammation is a complex immune reaction that includes both innate and adaptive immunity and can be caused by a variety of infectious agents. When the granuloma is caused by mycobacteria, disseminated disease has now been well documented using molecular methods. Molecular methods have also been very useful to define that not all granulomas with AFB are due to *M. tuberculosis *and that mycobacteria are sometimes found in the absence of bacilli. However, an etiology for granulomatous inflammation with or without necrosis is not always found, even with the use of molecular methods. Defining which granulomas need to be tested using molecular methods, which probes or primers and protocols to use routinely, and what is the ideal specimen is not clear at this point as the series presented vary largely in these aspects.

## Figures and Tables

**Table 1:** Etiologic causes of granulomas depending on the histopathologic characteristic.

Characteristic of granuloma	Description	Etiologic causes
With necrosis	Macrophages, multinucleated giant cells, lymphocytes, and central necrosis; calcifications may be present	Tuberculosis, chronic fungal infections (such as histoplasmosis, coccidioidomycosis, blastomycosis), Wegener's granulomatosis

With necrosis and eosinophils	Macrophages, multinucleated giant cells, lymphocytes, eosinophils, and necrosis	Parasites (including schistosomiasis, fasciolosis), sometimes some of the chronic fungal infections mentioned above

With stellate or geographic necrosis	Macrophages, multinucleated giant cells, lymphocytes, neutrophils, central necrosis that takes a stellate shape	Tularemia, bartonellosis, lymphogranuloma venereum, actinomycosis, chronic granulomatous disease

With suppurative inflammation	Macrophages, lymphocytes, abundant neutrophils, and different amounts of necrosis	Tularemia, listeriosis, acute fungal infections

With abundant plasma cells	Macrophages, multinucleated giant cells, lymphocytes, plasma cells, and different amounts of necrosis	Syphilis

Foamy macrophage aggregates	Foamy macrophages with minimal necrosis and other inflammatory cells	Atypical mycobacteria (MAI), Whipple disease, *Rhodococcus equi*, lepromatous leprosy

Epithelioid granuloma with minimal or no necrosis	Small, macrophages, multinucleated giant cells, lymphocytes	Leishmaniasis, sarcoidosis, lupus, hepatitis C

Epithelioid granuloma with minimal or no necrosis but presence of eosinophils	Small, macrophages, multinucleated giant cells, lymphocytes, eosinophils	Rejection after transplant, response to medications

Ill defined	Small groups of macrophages	Hodgkin's disease, metastasis

Lipid granulomas	Macrophages with lipid vacoules, lymphocytes, may have some necrosis including fibrin deposition	Lipid containing foods, mineral oils, reactions to medications, toxoplasmosis

Granulomas with a vacuole surrounded by fibrin	Macrophages, multinucleated giant cells, lymphocytes, neutrophils, lipid vacuole (clearing) surrounded by fibrin	Q fever, rarely: leishmaniasis, toxoplasmosis, cytomegalovirus, typhoid

**Table 2:** Examples of detection of nontuberculosis mycobacteiria in patients with granulomatous inflammation.

Case	Histology	Specimen	Primer	Mycobacteria	Reference
Lung cavity in a patient with hyperlipidemia and gout	Caseous necrosis, sarcoidal granuloma, positive AFB	Formalin-fixed, paraffin-embedded	*hsp65* gene followed by sequencing	*M. avium* complex and *M. kansasii *	[47]

Old cavitary tuberculosis (5 patients) and COPD (1)	Caseous necrosis, bronchiectasis (2 of 6 with positive AFB)	Formalin-fixed, paraffin-embedded	*hsp65* gene followed by sequencing	*M. avium* complex (one case with *M. tuberculosis*)	[47]

Masses in 2 HIV positive patients	Spindle cell pseudotumor with abundant AFB staining	Formalin-fixed, paraffin-embedded	*hsp65* gene followed by sequencing or restriction fragment polymorphisms	*M. avium-intracellulare *	[35]

Lymphadenitis in children	Caseous granulomas (1 of 2 with positive AFB)	Tissue	16S rRNA gene and the IS*1245 *specific sequence for *M. avium* subsp. *hominissuis* using quantitative PCR; in one case RFLP was added	*M. avium* subsp. *hominissuis *	[50]

Lymphadenitis in children	Necrotizing granulomas (3 of 22 positive with AFB)	Culture	Probe not specified	*M. avium* complex and *M. fortuitum *	[51]

Lymphadenitis (4 cases)	Necrosis, lymphohistiocytic cells including epithelioid macrophages and giant cells, neutrophils	Fine needle aspirate (frozen)	JB21 and JB22	*M. bovis *	[38]

Granulomatous prostatitis after biopsy in 12 patients	Suppurative necrotizing granulomas with foamy histocytes and eosinophils, positive AFB in 5	Culture in 8 patients	Random amplified polymorphic DNA-PCR	*M. abscessus *	[49]

Hemoptysis in a patient with a bronchiectasis due to previous tuberculosis	No tissue available for study but AFBs present in stputum	Culture	*rpoB* and *hsp65* and sequencing RFLP of *rpoB* gene	*M. abscessus *	[52]

5 patients with chronic skin lesions in extremities, recurrent, some thought to be sarcoid or swimming pool granulomas	Granulomas, 2 of them with positive AFB	Formalin-fixed, paraffin-embedded	16S rRNA gene then sequencing	*M. haemophilum *	[53]

Multiple, progressive, painless, erythematous, nodular skin lesions in patient with liver transplant	Suppurative granulomatous inflammation with numerous AFB	Tissue	PCR of the *hsp65 *gene then sequencing of product	*M. haemophilum *	[54]

Chronic inflammation of hand skin	Granulomatous inflammation, AFB positive	Formalin-fixed, paraffin-embedded	16S rRNA gene then sequencing	*M. marinum *	[53]

Multiple, progressive, painless, erythematous, ulcerated, nodular skin lesions in patient with liver transplant and diabetes	Florid histiocytic infiltrates with scattered multinucleated giant cells surrounded by lymphocytes (negative AFB).	Culture	PCR of the *hsp65 *gene then sequencing of product	*M. marinum *	[54]

Granuloma in eyelid	Granulomatous inflammation (corpus alienum); AFB not done	Formalin-fixed, paraffin-embedded	16S rRNA gene then sequencing	*M. gordonae *	[53]

Epithelial cyst	Granulomatous inflammation; AFB not done	Formalin-fixed, paraffin-embedded	16S rRNA gene then sequencing	*M. malmoense *	[53]

Pulmonary nodule with spontaneous pneumothorax	Granulomas with necrosis in lung tissue and faint staining AFB	Culture	16S rRNA PCR and sequencing	*M. heckeshornense *	[55]

Formalin-fixed, paraffin-embedded	16S 8–27 F primers and sequencing

Progressive enlargement of lung nodules in an afebrile patient with chronic obstructive pulmonary disease	Necrotizing granulomatous inflammation, numerous AFB present	Fresh tissue	ITS region of the rRNA gene, then sequencing	*M. chimaera *	[56]

Skin papules and nodules in patient with Hodgkin's lymphoma	Granulomas with AFB present	Culture	*rpoB* and *hsp65* and sequencing	Novel mycobacteria, proposed *M. shigaense *	[48]

Granulomas in various tissues including respiratory tract, lymph nodes, gastrointestinal and genitourinary tracts, bone, skin (no separation was given by mycobacteria)	Granulomas with and without caseous necrosis (presence of AFB in only 4 cases)	Formalin-fixed, paraffin-embedded	*hsp65* gene followed by sequencing or restriction fragment polymorphisms	*M. chelonae *(13 cases); * M. mucogenicum *(8); *M. fortuitum *(7); *M. peregrinum *(1); *M. gordonae *(3); *M. rhodesiae *(1)	[35]

Leprosy	Not described	Tissue sections	Peptide nucleic acid fluorescent in situ hybridization using MLEP primer	*M. leprae *	[39]

**Table 3:** Methods that have been used to define nonmycobacterial infectious agents that can cause granulomatous inflammation.

Disease	PCR	Serology	Culture	Visualization of organism in histology or other	Ref.
*Bartonella henselae *	From lesion, 12 of 14 (86%); in another series that studied 33 granulomatous lymphadenitis 7 cases positive.	23 of 23 positive.	Recovery in cultures approaches 50%.	Requires bacterial silver staining for visualization, was detected in 12 of 19 (63%).	[5, 51]

*Brucella *	From bone marrow aspirates, serum and blood. Can be used after antibiotics have been given.	Several methods with varying sensitivity (agglutination, ELISA).	Sensitivity depends on stage of disease and sample type, requires extended culture time as the bacteria grows slowly. Laboratory acquired cases can occur, careful handling is needed.	Requires IHC or immune-fluorescence for visualization.	[26, 57]

Tularemia	RT-PCR from blood and tissues in 39% of 101 patients. 14 of 15 cases in tissues.	Microagglutination, immunofluorescence for detection of IgG and IgM in 94% of 101 patients.	Culture in 21% of 101 patients.	Requires bacterial silver staining or IHC for visualization	[58, 59]

*Yersinia enterocolitica *	Not done.	ELISA testing is not useful.	Most cases diagnosed this manner. MALDI-FOF has been used for specific identification.	Not commented.	[51, 60, 61]

Syphilis	PCR 39 to 69% of biopsies.	Varies depending on stage.	Not available.	Requires bacterial silver stain (Dieterle or Steiner) for visualization (25%); increases sensitivity with IHC (49 to 51%).	[62, 63]

*Leishmania *	Used for detection and defining species, in meta-analysis very high diagnostic odds ratio. Using skin formalin-fixed, paraffin-embedded samples detection rate is 97%.	Limited sensitivity which depends on assay used, type of disease (cuteanous versus visceral).	Can be done but not routine with 58% sensitivity.	Visualization with H&E, but when numbers of parasites are low, they may be difficult to diagnose. Enhanced with IHC to 88% sensitivity.	[64–67]

*Toxoplasma *	Can be performed in tissues and blood, different primers, good for diagnosis of congenital disease.	Variety of assays available. IgG indicates past infection, IgM can remain increased up to 2 years after acute infection.	By inoculating mice (in reference laboratories).	Visualization with H&E in placenta and other tissues. Enhanced with IHC.	[68–70]

*Acanthamoeba *	Can reach 89% sensitivity in specimens from patients with keratitis. Cyst formation decreases sensitivity. Species can be defined using PCR.	Not available.	Is done primarily for diagnosis of keratitis using a lawn of *Escherichia coli. *	Amoeba are visualized with H&E in patients with granulomatous meningo encephalitis or keratitis; enhanced diagnosis by use of immune assays (DFA or IHC).	[71–74]

*Schistosoma *	PCR in urine can have up to 100% sensitivity and 91% specificity.	Detection of antibodies: may cross-react with other helminth infections, IgM may persist long after acute infection. Detection of antigen: in urine and serum. Sensitivity ranges from 41–78% and specificity between 76–100%.	Not available.	Detection of ova in stool or urine; quantification in a fixed amount of urine or stool allows to determine intensity of infestation. Adult worms and eggs can be seen in tissue sections.	[75–78]

*Fasciola *	PCR from stools and eggs from adult worms obtained from humans and animals.	By ELISA the sensitivity is 95% and specificity 95%. No correlation with number of ova in stool.	Not available.	Detection of ova in stool. Adult worms and eggs can be seen in tissue sections.	[79–81]

*Anisakis *	Case detected by PCR from ileocecal formalin-fixed, paraffin-embedded tissue.	Seroprevalence using IgE ELISA has been documented to be 6% in Korea.	Not available.	Visualized with H&E.	[82, 83]

*Pneumocystis jirovecii *	PCR has been used in bronchioalveolar lavage for diagnosis and to define colonization in transplant patients. Can be used for genotyping.	(1 → 3) *β*-D-glucan has a diagnostic sensitivity of 95% but specificity is 86% since there is cross-reactivity with other fungal infections.	Not available.	Visualized with H&E or fungal silver stain. Fluorescent antibodies can be used.	[12, 84–87]

*Histoplasma *	Sensitivity of PCR on formalin-fixed, paraffin-embedded tissues 89%. From fresh specimens sensitivity is 100% and specificity 73%.	Urine and serum antigen cross-react with *Blastomyces*. Antibodies can also be measured.	Can take up to 4 weeks to grow. Can be found in blood cultures.	Visualized with H&E, PAS and fungal silver stain (these are small yeasts with narrow based budding).	[84, 88, 89]

*Blastomyces *	From fresh specimens sensitivity is 99% and specificity 86%.	Urine and serum antigen cross-react with *Histoplasma*. Antibodies can also be measured.	Grows well but may take several weeks.	Visualized with H&E, PAS and fungal silver stain (these are 6–15 micron yeasts with broad based budding).	[84, 89, 90]

*Coccidioides *	PCR on fresh respiratory specimens has a sensitivity of 75 and specificity of 99%.	Antibodies can be measured using several methods. IgG response can be abrogated if treatment is started early.	Grows within 2 to 3 weeks. It is a select agent and it needs to be handled with care due to potential laboratory transmission.	Visualized with H&E, PAS and fungal silver stain (diagnostic structures are spherules with endospores; endospores on their own can be confused with *Blastomyces *and other yeasts).	[84, 91–93]

*Paracoccidioides *	PCR has been performed in tissues, including fine needle aspirates. There are commercial kits available.	Several methods available, including Western blots. Some have important cross-reactivity with other yeasts, particularly *Histoplasma*.	Growth may take up to 2 months.	Visualized with H&E, PAS and fungal silver stain (yeasts with multiple—more than 3 buds, mariner's wheel, are diagnostic).	[84, 94–96]

IHC: immunohistochemistry, H&E: hematoxylin and eosin, DFA: direct fluorescent antibody, PAS: periodic acid shift stain.
